# 4PBA reduces growth deficiency in osteogenesis imperfecta by enhancing transition of hypertrophic chondrocytes to osteoblasts

**DOI:** 10.1172/jci.insight.149636

**Published:** 2022-02-08

**Authors:** Amanda L. Scheiber, Kevin J. Wilkinson, Akiko Suzuki, Motomi Enomoto-Iwamoto, Takashi Kaito, Kathryn S.E. Cheah, Masahiro Iwamoto, Sergey Leikin, Satoru Otsuru

**Affiliations:** 1 Department of Orthopaedics, University of Maryland School of Medicine, Baltimore, Maryland, USA.; 2 Department of Orthopaedic Surgery, Osaka University, Graduate School of Medicine, Osaka, Japan.; 3 School of Biomedical Sciences, Li Ka Shing Faculty of Medicine, University of Hong Kong, Hong Kong, China.; 4 Section on Physical Biochemistry, Eunice Kennedy Shriver National Institute of Child Health and Human Development (NICHD), NIH, Bethesda, Maryland, USA.

**Keywords:** Bone Biology, Cell Biology, Cartilage, Cell stress, Genetic diseases

## Abstract

Short stature is a major skeletal phenotype in osteogenesis imperfecta (OI), a genetic disorder mainly caused by mutations in genes encoding type I collagen. However, the underlying mechanism is poorly understood, and no effective treatment is available. In OI mice that carry a G610C mutation in COL1A2, we previously found that mature hypertrophic chondrocytes (HCs) are exposed to cell stress due to accumulation of misfolded mutant type I procollagen in the endoplasmic reticulum (ER). By fate mapping analysis of HCs in G610C OI mice, we found that HCs stagnate in the growth plate, inhibiting translocation of HC descendants to the trabecular area and their differentiation to osteoblasts. Treatment with 4-phenylbutyric acid (4PBA), a chemical chaperone, restored HC ER structure and rescued this inhibition, resulting in enhanced longitudinal bone growth in G610C OI mice. Interestingly, the effects of 4PBA on ER dilation were limited in osteoblasts, and the bone fragility was not ameliorated. These results highlight the importance of targeting HCs to treat growth deficiency in OI. Our findings demonstrate that HC dysfunction induced by ER disruption plays a critical role in the pathogenesis of OI growth deficiency, which lays the foundation for developing new therapies for OI.

## Introduction

Osteogenesis imperfecta (OI) is a heritable bone dysplasia characterized by bone fragility, low bone mass, recurrent fractures, deformities of long bones and spine, and short stature (1). Over 85% of OI patients carry dominant autosomal mutations in genes encoding type I collagen (*COL1A1* and *COL1A2*), which is a major protein of the bone matrix. Recessive and X-linked OI mutations have also been identified in genes that encode proteins responsible for transcriptional regulation, posttranslational modifications, trafficking, and processing of type I collagen (1).

Although OI is known as a brittle bone disease, recent analyses of data from a substantial number of patients have demonstrated pronounced growth deficiency in patients with severe forms of OI (2, 3). Bisphosphonates are the current mainstay therapy for strengthening bones and reducing pain in OI, but their effects on longitudinal bone growth are minimal (4–9). Even growth hormone shows limited effects on bone growth in patients with OI (10–12). Presently, there is no effective treatment to address growth deficiency, mainly because the mechanism underlying impaired growth in OI remains unknown.

Type I collagen is a heterotrimer, consisting of two α1 and one α2 chains. Its procollagen precursor contains N- and C-terminal propeptides, which are cleaved during collagen maturation. Procollagen pro-α1 and pro-α2 chains assemble at C-terminal propeptides and fold toward the N-terminal in a zipper-like manner within the endoplasmic reticulum (ER). Its central triple helical domain contains repeats of the glycine-X-Y (Gly-X-Y) tripeptide, which are critical for tight helical folding (13). The majority of dominant mutations in severe OI are substitutions of the obligatory Gly in this repeating tripeptide, and they hinder triple helix formation and result in misfolding of type I procollagen. This misfolded mutant procollagen accumulates in the ER, causing ER lumen dilation and disruption of ER structure and function. The resulting cell stress leads to cellular dysfunction — for instance, impaired maturation of osteoblasts (14, 15). This osteoblast dysfunction induced by misfolded procollagen accumulation in the ER plays a critical role in pathogeneses of dominant forms of OI (16–18).

Longitudinal bone growth takes place via endochondral ossification in the growth plate where chondrocytes proliferate, produce matrix, and undergo progressive steps of maturation until they differentiate into late hypertrophic chondrocytes (HCs) at the chondro-osseous junction. Late HCs contribute to formation of primary spongiosa by mineralizing and degrading cartilage matrix, recruiting blood vessels, and inducing osteo/chondro-clastogenesis (19–21). Moreover, recent studies have demonstrated that HCs also contribute to conversion of primary spongiosa to trabecular bone by transitioning to type I collagen–expressing osteoblasts (22–25), suggesting that expression of mutant type I collagen may affect OI HCs during their transition to osteoblasts. Inhibition of these HC activities impairs completion of endochondral ossification and slows longitudinal bone growth (19, 26–32). Contrary to extensive investigation on OI osteoblasts, the OI growth plate has not been sufficiently studied. Previous studies showed that the growth plate of OI patients had increased thickness of the hypertrophic zone with reduced mineralization, alkaline phosphatase activity, and matrix glycosaminoglycans (33–35), suggesting that HC activities are somehow disturbed in OI. Recently, we confirmed significantly increased height of the growth plate, particularly the hypertrophic zone, in a murine OI model carrying a G610C mutation identified in OI patients from the Old Order Amish kindred (36). This mutation replaces Gly610 in the triple helical region of the α2 chain of type I procollagen with cysteine. (Here, we follow the traditional notation for Gly substitutions in OI, in which the Gly number is counted from the N-terminal end of the triple helix — i.e., triple helical Gly610 is Gly706 in the full-length pro-α2 chain). We have demonstrated that late HCs adjacent to the chondro-osseous junction express type I collagen and that the ER cisternae of late HCs in G610C OI mice are significantly dilated compared with those in WT littermates, suggesting that expression of mutant type I collagen causes cell stress in OI late HCs. In vitro chondrocyte pellet culture showed that maturation of OI HCs was significantly suppressed, and it showed increased expression of cell stress–related genes. These findings suggest that HC dysfunction caused by ER disruption may play an important role in growth deficiency of dominant OI (37). Of note, it has been demonstrated that ER disruption in osteoblasts does not affect bone length, while that in HCs leads to dwarfism (38–44), also supporting the possibility that HC dysfunction is a critical contributor to impaired growth in dominant OI.

ER disruptions caused by accumulation of misfolded procollagen with Gly substitutions have been shown to activate different cell stress pathways with or without involvement of canonical unfolded protein response (UPR) (17, 45). General cell response to stress (integrated stress response [ISR]) involves increased phosphorylation of eIF2α by PERK (in UPR) or other kinases (in other pathways), and this causes overall downregulation of mRNA translation that reduces the ER workload (46, 47). Other features, which include increased expression of various ER chaperones, ER-associated degradation (ERAD) of misfolded proteins by proteasomes, and autophagy, are more dependent on the specific cell stress pathway. Regardless of the cell stress pathway, however, ER dilation indicates that the accumulation of misfolded proteins in the ER exceeds the capacity of ISR, ERAD, and autophagy, in which case cells undergo apoptosis or become dysfunctional. 4-Phenylbutyric acid (4PBA), a drug approved by the US Food and Drug Administration for urea cycle disorders, is known to reduce misfolded protein accumulation in the ER (48). Hence, it is sometimes referred to as a chemical chaperone, although the mechanism of its action is not fully understood. It has been demonstrated that 4PBA enhances protein clearance from the ER and reduces ER dilation in cultured fibroblasts isolated from patients with dominant and recessive OI (49, 50). Moreover, 4PBA improves bone mineralization and deformities by reducing ER disruption in the *Chihuahua* zebrafish model of OI (51).

We have shown previously that maturation of HCs derived from G610C OI mice was significantly suppressed (37). In this study, using G610C OI mice, we sought to investigate if HC dysfunction induced by ER disruption plays a critical role in the pathogenesis of OI growth deficiency and whether reduction of ER disruption with 4PBA can improve impaired growth by rescuing HC dysfunction.

## Results

### Cell turnover in the growth plate is significantly reduced in G610C OI mice.

**Figure 1:**
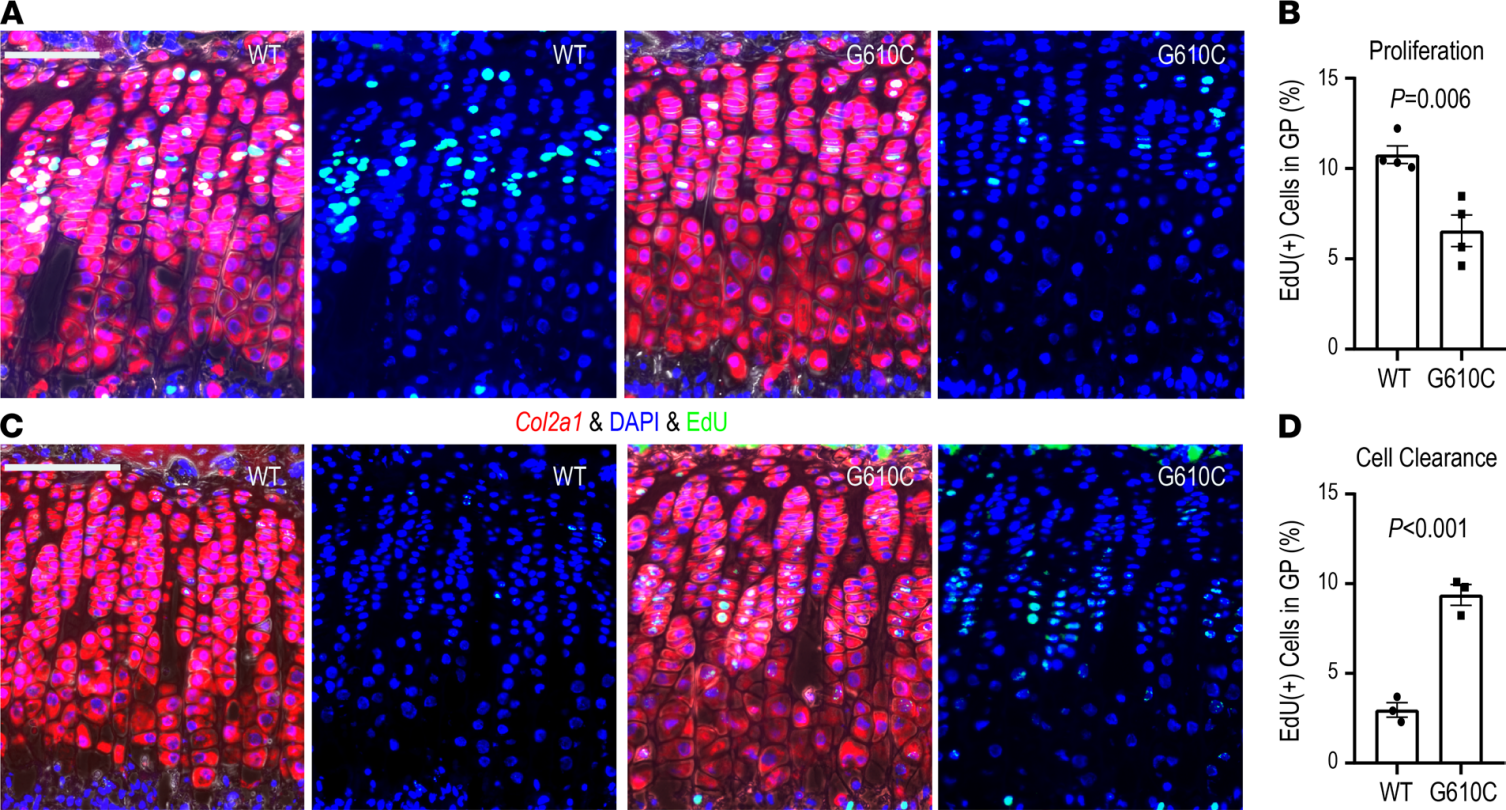
Cell turnover in the growth plate is significantly reduced in G610C OI mice. (**A**) Representative images of EdU staining combined with in situ hybridization for *Col2a1* in the growth plate from G610C OI mice (G610C) and WT littermates (WT) 24 hours after EdU administration (red, *Col2a1*; green, EdU; and blue, DAPI). Scale bar: 100 μm. (**B**) Percentage of EdU-labeled chondrocytes in the growth plate (*n* = 4 mice per group, *P* = 0.006, 2-tailed Student’s *t* test). (**C**) Representative images of EdU staining combined with in situ hybridization for *Col2a1* in the growth plate 4 days after EdU administration (red, *Col2a1*; green, EdU; and blue, DAPI). Scale bar: 100 μm. (**D**) Percentage of EdU-positive chondrocytes in the growth plate (*n* = 3 mice per group, *P* < 0.001, 2-tailed Student’s *t* test). Data are shown as mean ± SEM.

As we recently reported, G610C OI mice have significantly shorter bones compared with the WT littermates. Additionally, histological analyses revealed that the height (thickness) of the growth plate, particularly the height of the hypertrophic zone, was significantly increased in G610C OI mice (37), suggesting that chondrocytes stagnate in the growth plate. To examine chondrocyte proliferation in the growth plate, we i.p. injected 5-ethynyl-2′-deoxyuridine (EdU) into 4-week-old G610C OI mice and their WT littermates and harvested tibiae 24 hours after the injection. Chondrocytes were identified by in situ hybridization for *Col2a1* on the tibial sections, followed by EdU staining, with DAPI counter staining. The percentage of chondrocytes incorporating EdU in the entire growth plate of G610C OI mice was significantly less than that of WT littermates (Figure 1, A and B), indicating that chondrocyte proliferation in the growth plate is suppressed in G610C OI mice.

To evaluate cell turnover in the growth plate, EdU was i.p. injected into mice in 2 consecutive days at 3 weeks of age, and the percentage of EdU-positive chondrocytes in the growth plate was examined 4 days after the EdU injection. Consistent with previous reports showing that chondrocytes pass through the hypertrophic zone within 48 hours (23, 52), WT littermates had very few EdU-positive chondrocytes in the growth plate. In contrast, approximately 10% of EdU-positive cells remained in the growth plate of G610C OI mice (Figure 1, C and D), suggesting that chondrocyte turnover in the growth plate is significantly reduced in G610C OI mice.

### HCs stagnate in the growth plate in G610C mice.

**Figure 2:**
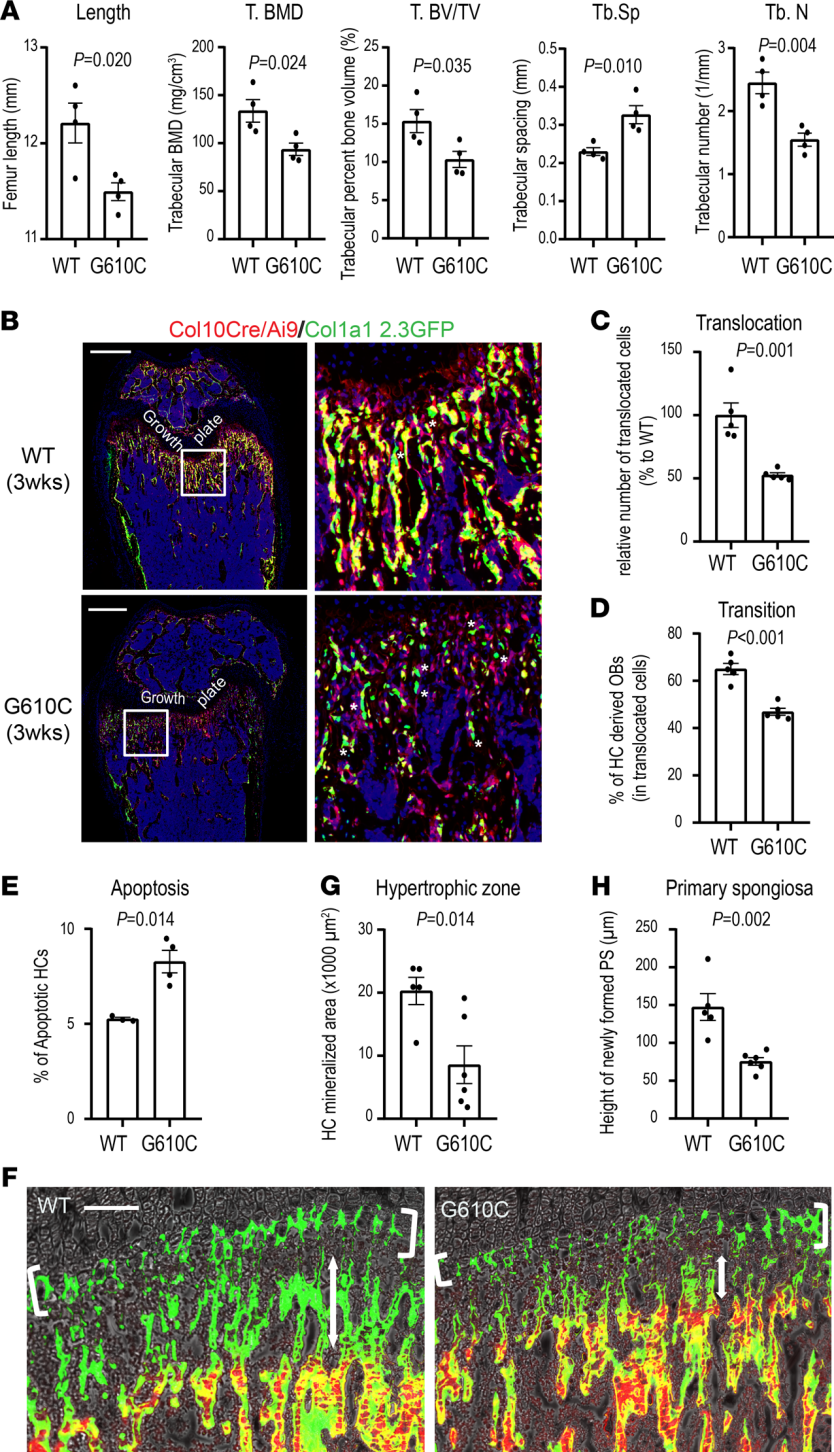
Hypertrophic chondrocytes stagnate in the growth plate in G610C mice. (**A**) μCT analysis of femora from female G610C OI mice and female WT littermates at 3 weeks of age (*n* = 4 mice per group, 2-tailed Student’s *t* test). (**B**) Representative images of femoral sections from Col10a1-Cre;Ai9;Col1a1 2.3-GFP and Col10a1-Cre;Ai9;Col1a1 2.3-GFP;G610C mice at 3 weeks of age. The white boxes are enlarged in the right panels. Green osteoblasts are indicated by white asterisks. Scale bars: 500 μm. (**C**) Percentage of HC-derived cells (red + yellow cells) in the trabecular area relative to those in Col10a1-Cre;Ai9;Col1a1 2.3-GFP mice in **B** (*n* = 5 mice per group, *P* = 0.001, 2-tailed Student’s *t* test). (**D**) Percentage of HC-derived osteoblasts (100% × [yellow cells]/[red + yellow cells]) in the trabecular area in **B** (*n* = 5 mice per group, *P* < 0.001, 2-tailed Student’s *t* test). (**E**) Percentage of TUNEL-positive apoptotic cells in HCs (WT, *n* = 3 mice; G610C, *n* = 4 mice; *P* = 0.014; 2-tailed Student’s *t* test). (**F**) Representative images of the tibial growth plate/primary spongiosa area labeled with calcein and alizarin red. Scale bar: 100 μm. (**G**) Quantification of mineralized area in the hypertrophic zone indicated by white brackets in **F** (WT, *n* = 5 mice; G610C, *n* = 6 mice; *P* = 0.014; 2-tailed Student’s *t* test). (**H**) Height of newly formed primary spongiosa during the interval between calcein and alizarin red injections (47 hours) indicated by double arrows in **F** (WT, *n* = 5 mice; G610C, *n* = 6 mice; *P* = 0.002; 2-tailed Student’s *t* test). Data are shown as mean ± SEM.

As we previously demonstrated (37), late HCs adjacent to the chondro-osseous junction in G610C mice have dilated ER, raising a question of whether HCs are dysfunctional in G610C OI mice. HCs, especially late HCs, are known to play an important role in endochondral ossification at the chondro-osseous junction. Recent studies show that the transition of some HCs to osteoblasts contributes to bone formation in the trabecular area (22–24). The importance of proper transition of HCs to the subchondral bone is illustrated by the impact of inactivation of Wnt signaling and IRX3/5 transcription factors in the HC lineage. Conditional deletion of β-catenin specifically in HCs reduced transition of HCs and resulted in reduced trabecular bone formation with shorter bone length (29). Inactivation of *Irx3* and *Irx5* in the HC lineage compromises osteogenic fate decision, leading to reduced trabecular bone and increased adipogenesis (53). These studies suggest that HC transition to osteoblasts is critical for trabecular bone formation and longitudinal bone growth. μCT analysis of femora from G610C OI mice and WT littermates at 3 weeks old showed significantly reduced trabecular bones and shorter bone length in G610C OI mice (Figure 2A). Therefore, we tracked HC lineage cells in a potentially novel strain derived from Col10a1-Cre–knock-in mice (23) and Ai9 reporter mice that express tdTomato — a variant of red fluorescent protein — following Cre-mediated recombination (54): Col1a1 2.3-GFP mice that express GFP in mature osteoblasts and osteocytes (55) and G610C OI mice (Col10a1-Cre;Ai9;Col1a1 2.3-GFP;G610C mice). Mice without the G610C mutation served as WT (non-OI) controls (Col10a1-Cre;Ai9;Col1a1 2.3-GFP mice). In these animals, HCs and their descendent cells express red fluorescence, and mature osteoblasts/osteocytes express green fluorescence. Mature osteoblasts/osteocytes that are derived from HCs express both fluorescence markers and appear yellow. We verified that a substantial number of HC-derived cells survived in the trabecular area in 3-week-old Col10a1-Cre;Ai9;Col1a1 2.3-GFP mice, shown as red and yellow cells, consistent with their lineage origin (Figure 2B). Some of red cells became mature osteoblasts/osteocytes and appeared as yellow cells. Interestingly, the number of cells derived from HCs (red and yellow cells) translocated from the growth plate to the metaphysis was significantly reduced in G610C OI mice compared with WT controls (Figure 2C). The transition of HCs to osteoblasts (percentage = yellow/[yellow + red]) in the trabecular area was also significantly decreased in G610C OI mice (Figure 2D). Notably, a significantly higher number of green mature osteoblasts/osteocytes were observed — particularly in the trabecular area of G610C OI mice, indicating that osteoblasts, presumably derived from the perichondrium, were partially compensating for the lack of osteoblasts derived from the growth plate.

In addition, TUNEL staining revealed that the G610C OI growth plate had shown a slight but significant increase in apoptosis (Figure 2E), which also contributes to the reduction in the HC translocation from the growth plate to the metaphysis in G610C OI mice. These findings indicate that impaired transition of G610C OI HCs from the growth plate to subchondral bone reduces the supply of osteoblasts from the growth plate. It seems that the increase in osteoblast recruitment from the perichondrium was not sufficient to fully compensate for this reduction in osteoblast supply from the growth plate, resulting in significantly less trabecular bone formation in G610C OI mice.

### The primary spongiosa formation is impaired in G610C OI mice.

To further evaluate and visualize the contribution of the mutant HCs to endochondral ossification, mineralization in the growth plate, and primary spongiosa, alizarin complexone was injected 48 hours and calcein 1 hour prior to euthanasia to 3-week-old G610C OI mice and their WT littermates. This experiment visualized a mineralized HC layer (Figure 2F, white brackets) and a primary spongiosa layer (Figure 2F, double arrows) formed between the injections as regions labeled only with calcein. The mineralized area in the hypertrophic zone and the height of newly formed calcified cartilage were significantly reduced in G610C OI mice (Figure 2, G and H), suggesting that, in addition to osteoblast dysfunction, HC dysfunction contributes to reduced trabecular bone formation.

### 4PBA treatment improved HC transition/translocation in G610C OI mice.

**Figure 3:**
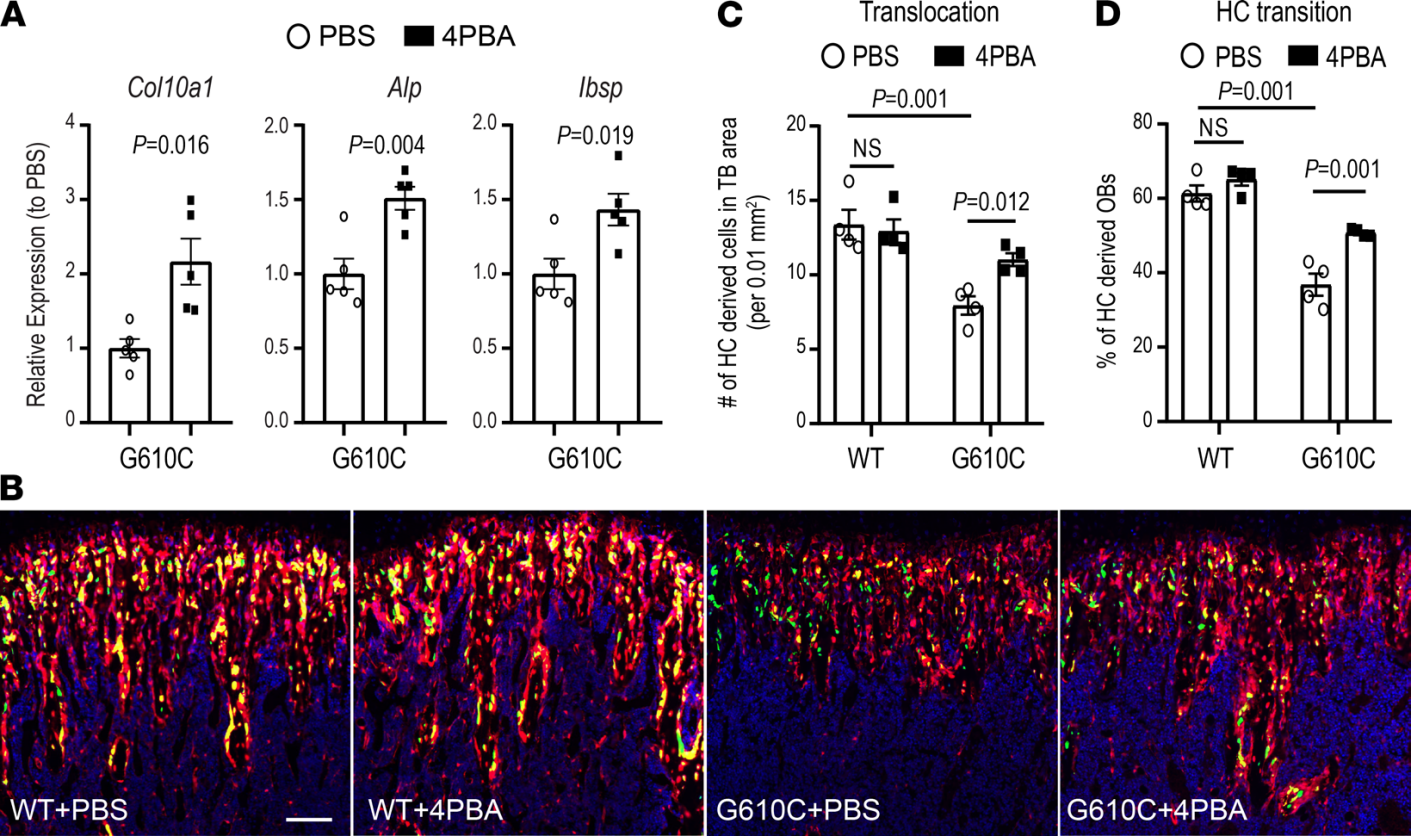
4PBA treatment improves translocation and osteoblastic transition of hypertrophic chondrocyte in G610C OI mice. (**A**) Effect of overnight treatment with 4PBA (5 mM, filled square dots) on *Col10a1*, *Alp*, and *Bsp* mRNA in G610C chondrocyte pellets cultured for 3 weeks relative to the mean values in the control group treated with PBS (circle dots, *n* = 5 per group, 2-tailed Student’s *t* test). (**B**) Representative images of the trabecular area of tibiae from 3-week-old Col10a1-Cre;Ai9;Col1a1 2.3-GFP and Col10a1-Cre;Ai9;Col1a1 2.3-GFP;G610C mice treated with either PBS or 4PBA (0.4 mg per day) for 10 days. Scale bar: 100 μm. (**C**) The number of HC-derived cells (red + yellow cells) per 0.01 mm^2^ in the trabecular area (*n* = 4 per group; 2-way ANOVA; treatment-genotype interaction, *P* < 0.05). (**D**) The percentage of osteoblasts (yellow cells) in HC-derived cells (red + yellow cells) in the trabecular area (*n* = 4 per group; 2-way ANOVA; treatment-genotype interaction, *P* < 0.05). Data are shown as mean ± SEM.

As we reported previously, HCs in the G610C OI growth plate express mutated type I collagen, resulting in ER dilation and cell stress (37). To determine whether HC dysfunction induced by ER disruption is responsible for the impaired translocation of HCs to the primary spongiosa, we treated chondrocytes in pellet culture with 4PBA, a chemical chaperone that facilitates protein folding. Chondrocyte pellet culture mimics hypertrophic differentiation in the growth plate. After 3 weeks in pellet culture, chondrocytes become hypertrophic and increase expression of hypertrophy-related genes such as *Col10a1*, *Alp*, and *Ibsp* (56). Our previous report demonstrated that the expression levels of hypertrophy-related genes at 3 weeks were significantly lower in G610C OI chondrocyte pellets compared with those in WT chondrocyte pellets (37). Treatment of G610C OI chondrocyte pellets with 4PBA for 24 hours significantly increased the expression levels of the hypertrophy-related genes (Figure 3A), suggesting that reduction of cell stress rescued the suppressed maturation of G610C OI chondrocytes.

We then treated 3-week-old Col10a1-Cre;Ai9;Col1a1 2.3-GFP;G610C mice with a daily injection of 4PBA (0.4 mg) for 10 days to examine if the reduction of cell stress in HCs improves their osteoblastic transition and translocation. Consistent with our findings in Figure 2B, the number of cells derived from HCs in the trabecular area, as well as the number of osteoblasts derived from HCs after PBS treatment, were significantly lower in mice carrying the G610C mutation compared with control littermates (Figure 3, B–D). 4PBA treatment did not affect WT HCs, but it significantly improved the osteoblastic transition and translocation of G610C HCs (treatment-genotype interaction *P* < 0.05), though not quite to the WT level (Figure 3, B–D). These results suggest that G610C OI HCs become dysfunctional due to cell stress, resulting in impaired HC to osteoblast lineage progression.

### Four-week treatment with 4PBA improved bone length and trabecular bone formation in G610C OI mice.

Since 10-day treatment improved HC function, we proceeded with daily injection of the same amount of 4PBA (0.4 mg) into G610C OI mice and their WT littermates from 3–7 weeks to determine whether ER disruption in HCs is a causal mechanism underlying growth deficiency in OI. We examined the effects of 4PBA on both female and male mice. While females and males had approximately the same body weight at 3 weeks, males gained more weight during the treatment course and received lower relative dosage of 4PBA as a result. Therefore, below, we focus on our observations for the female cohort, while the results for males are shown in Supplemental Figures 1 and 2 (supplemental material available online with this article; https://doi.org/10.1172/jci.insight.149636DS1).

**Figure 4:**
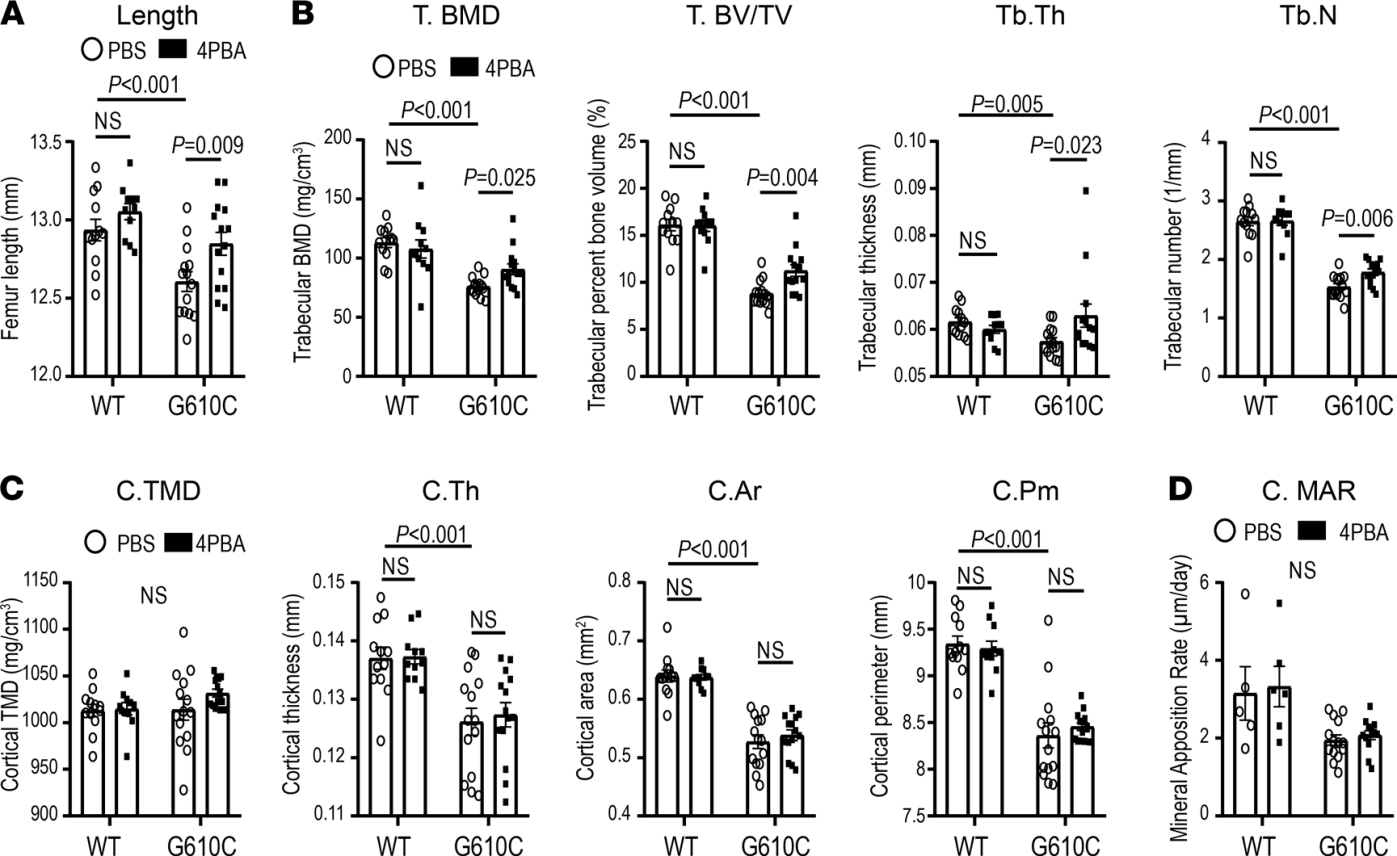
Four-week treatment with 4PBA improves bone length and trabecular bone formation in female G610C OI mice. (**A**–**C**) Femur geometry (μCT) in female G610C OI mice and female WT littermates (WT) treated by daily injections of PBS (circle dots) or 0.4 mg 4PBA in PBS (filled square dots) for 4 weeks (WT + PBS, *n* = 12; WT + 4PBA, *n* = 11; G610C + PBS, *n*=14; G610C + 4PBA, *n* = 14). (**A**) Femur length (2-way ANOVA; treatment-genotype interaction, *P* > 0.1). (**B**) Trabecular bone mineral density (T. BMD; 2-way ANOVA; treatment-genotype interaction, *P* < 0.05), trabecular bone volume fraction (T. BV/TV; Mann-Whitney *U* test), trabecular thickness (Tb. Th; Mann-Whitney *U* test), and trabecular number (Tb. N; 2-way ANOVA; treatment-genotype interaction, *P* < 0.1). (**C**) Cortical tissue mineral density (C. TMD; 2-way ANOVA; treatment-genotype interaction, *P* > 0.1), cortical thickness (C. Th; 2-way ANOVA; treatment-genotype interaction, *P* > 0.1), cross-sectional area (C. Ar; 2-tailed Student’s *t* test) and perimeter (C. Pm; 2-tailed Student’s *t* test). (**D**) Mineral apposition rate (MAR) at posterolateral periosteal surface (mid-diaphysis) per day (2-tailed Student’s *t* test). Data are shown as mean ± SEM. Mann-Whitney *U* or 2-tailed Student’s *t* test was used instead of 2-way ANOVA in **B**–**D** because of failure of normality or equal variance tests, respectively.

Female G610C OI mice treated with 4PBA for 4 weeks showed a significant improvement in the femur length compared with PBS-treated G610C OI controls, while 4PBA treatment had no significant effect on femur length in the control littermates (Figure 4A). Further analysis with μCT showed that all tested parameters related to trabecular bones such as trabecular bone mineral density (T. BMD), bone volume fraction (T. BV/TV), trabecular thickness (Tb. Th), and trabecular number (Tb. N) were significantly improved by 4PBA treatment, although these parameters were still inferior to those of WT littermates (Figure 4B). 4PBA treatment did not affect these parameters in the control littermates. In contrast, the tested parameters related to cortical bones including cortical tissue mineral density (C. TMD), cortical thickness (C. Th), cortical cross-sectional area (C. Ar), and cortical perimeter (C. Pm) were not affected by 4PBA treatment (Figure 4C). Dynamic histomorphometry showed that mineral apposition rate on cortical bone was not affected, either (Figure 4D). These results indicate that our 4PBA treatment enhanced bone length by improving HC function but not osteoblast function.

### 4PBA treatment did not improve bone strength.

**Figure 5:**
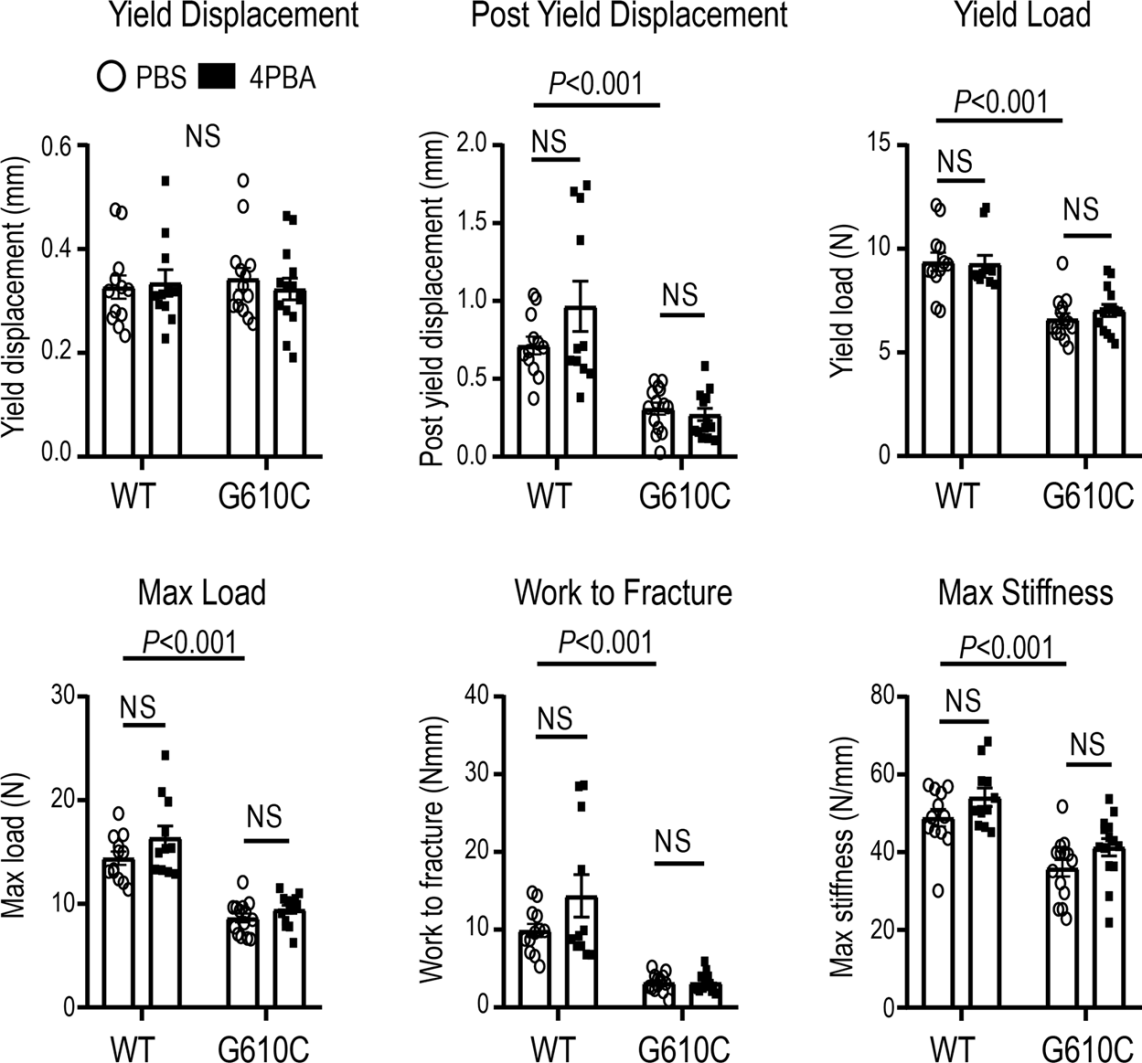
4PBA treatment does not improve bone strength. Effect of daily injections of PBS (circle dots) or 0.4 mg 4PBA in PBS (filled square dots) for 4 weeks on biomechanical parameters of the same femora as in Figure 4 (4-point bending test at mid-shaft). Data are shown as mean ± SEM. Two-way ANOVA was used for max stiffness (treatment-genotype interaction, *P* > 0.1); Mann-Whitney *U* test was used for yield load, yield displacement, and work to fracture (failed normality test); and 2-tailed Student’s *t* test was used for post yield displacement and max load (failed equal variance test).

To test whether 4PBA treatment ameliorates bone fragility, we performed a 4-point bending test on the midshaft of femora that were utilized for μCT analysis. G610C OI femora were significantly less stiff yet more brittle compared with those from WT littermates (Figure 5). 4PBA treatment had no significant effect on any of the biomechanical parameters such as yield displacement, postyield displacement, yield load, maximum load, work to fracture, and maximum stiffness (Figure 5). In addition to cortical mineralization and geometry, the strength of cortical bone at mid-diaphysis was unaffected by the 4PBA treatment.

### 4PBA treatment reduced ER dilation in HCs but not in osteoblasts.

**Figure 6:**
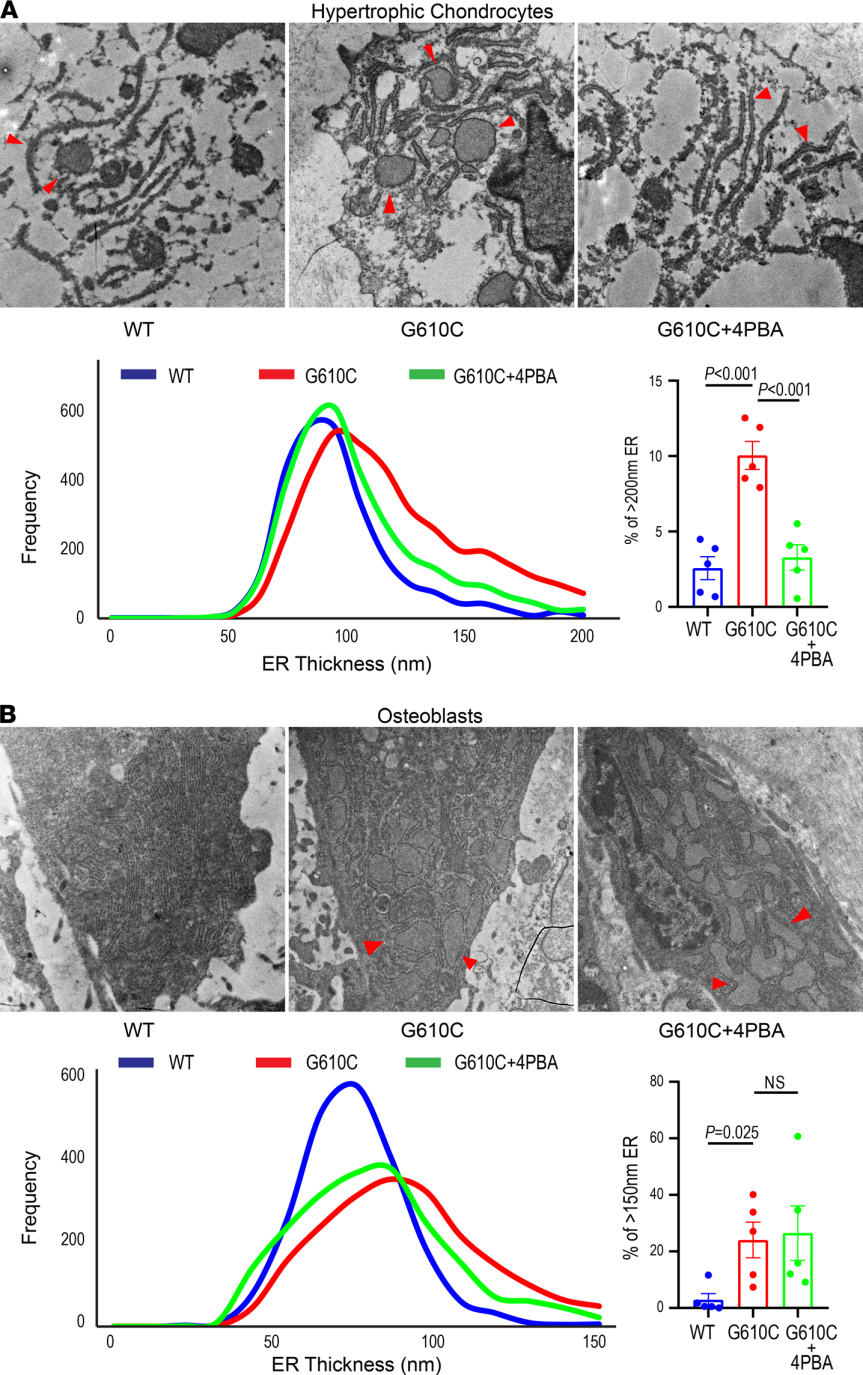
4PBA treatment improves ER dilation in hypertrophic chondrocytes but to a lesser extent in osteoblasts. (**A** and **B**) Representative transmission electron microscopic (EM) images of endoplasmic reticulum (ER, red arrowheads) in late hypertrophic chondrocytes from the tibial growth plate (**A**) and in tibial trabecular osteoblasts (**B**) after daily injections of PBS or 0.4 mg 4PBA in PBS (same animals as in Figures 4 and 5). Histograms show treatment effects on the ER thickness. Bar charts (mean ± SEM) show fractions of severely dilated ER in each mouse (≥ 200 nm thickness in HCs, ≥ 150 nm thickness in osteoblasts). ER morphology was examined by EM in *n* = 5 WT + PBS, *n* = 5 G610C + PBS, and *n* = 5 G610C + 4PBA animals from each of the groups selected based on their size being close to the mean value. We utilized the 2-tailed Student’s *t* test rather than 1-way ANOVA because meaningful statistical comparison could be performed only between WT + PBS and G610C + PBS animals (genotype effect) or between G610C + PBS and G610C + 4PBA animals (treatment effect), but not between WT + PBS and G610C + 4PBA animals.

To determine whether 4PBA improved HC function by reducing ER disruption, we examined HCs in the tibial growth plate from female mice treated with either 4PBA or PBS for 4 weeks using transmission electron microscopy. Because significant 4PBA effects on bone length and trabecular architecture were observed only in females, males were not examined. ER thickness was measured at 500 nm intervals in 5–12 HCs per growth plate (9–199 measurements per cell) and analyzed as previously described (37). The histogram of ER thickness in HCs of G610C OI mice (Figure 6A) was shifted to the right compared with that of WT littermates (Figure 6A), suggesting increased ER dilation in HCs from G610C OI mice. The fraction of severely dilated ER with ≥ 200 nm thickness was significantly greater in G610C OI than in WT mice (Figure 6A). Four-week treatment with 4PBA (Figure 6A) shifted the histogram of G610C OI mice to the left compared with untreated animals but did not fully rescue ER dilation compared with WT littermates. The fraction of severely dilated ER was decreased by the 4PBA treatment almost to the level observed in WT HCs (Figure 6A). In contrast, the 4PBA treatment had only a minimal effect on trabecular osteoblast ER (Figure 6B). Similar to HCs, the histogram of ER thickness was shifted to the right, and ER dilation with ≥ 150 nm thickness was more prominent in trabecular osteoblasts from G610C OI mice compared with WT littermates (21–163 measurements/cell, 5–9 cells/bone). The 4PBA treatment shifted the histogram slightly to the left, but the ER fraction with ≥ 150 nm thickness was not significantly changed (Figure 6B). Spatial transcriptomic analysis confirmed alleviation of cell stress and normalization of a transcriptional profile in HCs by 4PBA but showed a weaker effect on the cell stress and transcriptional profile of osteoblasts (Supplemental Figure 3). Apparently, 4PBA treatment had a significantly stronger effect on HCs than osteoblasts. These findings explain the distinct effects of 4PBA on bone growth and bone strength shown in Figures 4 and 5.

## Discussion

Short stature has been recognized as a skeletal manifestation commonly observed in patients with moderate to severe OI (2, 3). However, compared with extensive studies of bone fragility, the short stature aspect of OI has not been sufficiently studied, and its underlying mechanism remains unknown. As a result, there is presently no effective treatment to stimulate skeletal growth. Previous reports have demonstrated hypertrophic zone abnormalities in the growth plates of OI patients, such as increased height, lower mineralization, and reduced alkaline phosphatase activity (33–35). These observations suggest that HCs are affected in OI, although mechanisms of these effects have not been identified. Here, we report for the first time to our knowledge that ER disruption in late HCs inhibits their transition to osteoblasts, which results in HC stagnation in the growth plate in G610C OI mice, leading to growth deficiency. Reduction of ER disruption with chemical chaperone 4PBA releases HCs from the growth plate and enhances longitudinal bone growth.

Currently available therapies for OI are mainly aimed to strengthen bones. Antiresorption agents such as bisphosphonates and an antibody for receptor activator of NF-κB ligand (RANKL) have been demonstrated to have positive impacts on bone mineral density and bone strength but little effects on height (4–9, 57, 58). These results suggest that osteoclasts may not be responsible for growth deficiency in OI. Moreover, sclerostin antibody, an anabolic agent that stimulates osteoblast activity, has no effect on the bone length despite increasing bone mass and strength in multiple murine models of OI (59–61). Although osteoblasts are known to affect chondrocyte activities in the growth plate (62, 63), correction of osteoblast function failed to improve bone length in OI mice. Similarly, correction of overactive TGF-β signaling with neutralizing TGF-β antibody ameliorates osteopenia (64, 65) but does not increase bone length or improve callus formation during fracture healing in murine models of OI (64, 66), even though TGF-β regulates chondrocyte differentiation (67). Taken together, these and our observations suggest that HC dysfunction is a cell-autonomous effect of ER disruption in HCs rather than a consequence of defects in other cell types or bone environment, indicating the significance of targeting HCs to treat impaired growth in OI.

Consistent with previous reports (22–24), our fate mapping of HCs demonstrated that HC-derived cells survived in the trabecular area and that a substantial number of these cells became mature osteoblasts/osteocytes in growing WT mice (Figure 2B). The rate of transition of HCs into osteoblasts (Figure 2, C and D) and primary spongiosa formation (Figure 2H) was significantly reduced in growing G610C OI mice compared with WT, resulting in lower trabecular volume and number and in increased trabecular spacing (Figure 2A). Amelioration of HC translocation and transition to osteoblasts, as well as increased primary spongiosa formation, were likely responsible for the improvement in longitudinal bone growth and μCT trabecular bone parameters after the 4PBA treatment (Figure 4, A and B).

Previous studies of 4PBA as a potential therapeutic agent in OI focused on improving osteoblast function rather than HC-to-osteoblast transition. Using in vitro culture of fibroblasts from dominant and recessive OI patients as a proxy, type I procollagen was shown to accumulate in the ER, leading to ER enlargement and cell stress (49, 50, 68). 4PBA ameliorates such ER disruption, reduces cell stress, and improves cellular function by facilitating protein secretion and autophagy as a chemical chaperone and histone deacetylase inhibitor (49, 50, 69). However, the ER disruption and cell stress response to it vary depending on the specific OI mutation (49, 50, 68), which might contribute to differences in the response to treatments between patients harboring distinct mutations (1, 12, 70). Like other treatments, 4PBA may also have variable effects, depending on how it is administered, the mutation, and cell type, resulting in distinct 4PBA treatment effects we observed.

In our study, 4PBA treatment of young mice improved the trabecular bone formation and longitudinal bone growth, but not cortical bone formation, geometry, or strength (Figures 4 and 5). These effects were consistent with the longitudinal bone growth and trabecular bone being dependent primarily on HC-to-osteoblast transition, with bone strength and cortical bone being dependent primarily on osteoblast function. Indeed, HC-derived osteoblasts do not contribute to the outer cortical bone, in which osteoprogenitors differentiate directly to osteoblasts (23, 71). These results emphasize the necessity of targeting HCs rather than osteoblasts to treat short stature in OI.

Spatially resolved transcriptomic (SRT) analysis provided supplementary evidence supporting different types of cell stress and different 4PBA effects on HCs and osteoblasts (Supplemental Figure 3). While full interpretation of these experiments is beyond the scope of the present paper and will be reported elsewhere, several most relevant findings are worthy of a brief discussion. Most importantly, we confirmed that the G610C mutation had different effects on cell stress genes in HCs and osteoblasts (Supplemental Figure 3C). Type I collagen triple helix misfolding caused by this mutation was previously shown to cause noncanonical cell stress without UPR in osteoblasts (17), yet it induced UPR in HCs (37). Consistently, SRT analysis revealed cell stress involving increased *Hspa5* (BIP) expression in HCs and cell stress without increased *Hspa*5 expression in osteoblasts (Supplemental Figure 3C). One possible explanation of this effect is a much lower fraction of type I collagen and a much higher fraction of globular proteins in the ER of HCs compared with osteoblasts (Supplemental Figure 3D). On its own, collagen triple helix misfolding does not sequester the master regulator of UPR, BIP (45), explaining why it does not trigger UPR in osteoblasts (17). However, the resulting ER disruption (Figure 6) could result in misfolding of globular proteins, causing BIP sequestration and UPR as a secondary response in HCs (72). For instance, ER disruption by the mutation in HCs could affect folding of type II, IX, X, and XI collagen; martrilin-3; cartilage oligomeric matrix protein (COMP); and other matrix proteins known to cause cell stress in chondrocytes in various cartilage disorders (40, 43, 44, 73, 74). Synthesis of much less type I collagen (Supplemental Figure 3D) and a much higher fraction of secretory globular proteins could explain the more pronounced secondary UPR in HCs compared with osteoblasts (supported by increased expression of *Hspa5* and *Hsp90aa1* in HCs but not osteoblasts). The bigger role of this secondary UPR and its alleviation could then explain a more pronounced 4PBA effect on reducing ER dilation (Figure 6), restoring the transcriptomic profile (Supplemental Figure 3B), and normalizing the function of HCs compared with osteoblasts (Figures 4 and 5). In addition to different effects on different types of cell stress, rescue of osteoblasts that express much more type I collagen for a much longer time than HCs might require a different 4PBA treatment regimen. Daily injections of short-living 4PBA (approximately 0.8-hour half-life in blood [DRUGBANK; https://www.drugbank.ca/drugs/DB06819]) might be sufficient for alleviating ER disruption and rescuing HC function and transition to osteoblasts. The alleviation of ER disruption and rescue of osteoblast function by 4PBA might be possible, as well, but it might require higher 4PBA dosage and higher injection frequency.

It is worth noting that 4PBA significantly improved bone length and trabecular parameters in female G610C OI mice (Figure 4, A and B), while 4PBA effects on the same parameters in male mice were not statistically significant (Supplemental Figure 1, A and B). In addition to the effects of estrogen hormones, one possible explanation is that the administered fixed amount (0.4 mg) of 4PBA per injection was not sufficient for males. There was not much difference in body weight between 3-week-old male and female mice at the start of the treatment. However, males gained more weight during the treatment course and, therefore, received less 4PBA per gram of body weight than females, which could contribute to weaker 4PBA effects. Another possible explanation is the inherent difference in the responses of female and male mice to OI mutations and, thereby, to 4PBA treatment targeting these responses. Indeed, effects of OI mutations on bone structure, mechanical properties, and fracture healing were shown to be sex dependent in oim/oim*, Crtap-*KO, and G610C mouse models (66, 75), consistent with our observations in 7-week-old G610C animals (compare Figures 4 and 5 with Supplemental Figures 1 and 2, respectively). Finally, different effects of 4PBA on female and male mice could also be related to sex-dependent differences in the timing of skeletal growth and maturation; these differences have been shown to become significant after 3 weeks of age (76). Therefore, not only the dosage, but also timing and duration of the treatment may need to be adjusted depending on sex. More detailed analysis of the sex-dependent effects requires further investigation of these multiple factors, which is beyond the scope of the present work.

Our study focuses on a model of OI caused by qualitative defects of type I collagen. However, a significant fraction of OI is caused by quantitative defects such as null allele mutations in *COL1A1* causing type I OI. In this case, 4PBA treatment is not likely to be beneficial, since the latter mutations are not expected to cause cell stress or alter collagen clearance from cells.

Overall, our study demonstrates that cell stress caused by the accumulation of mutant type I collagen in the ER inhibited HCs from exiting from the growth plate. Reduction of cell stress using 4PBA rescued this inhibition of HC proceeding to osteoblast and HC translocation, resulting in enhanced longitudinal bone growth. These results indicate that ER disruption in HCs may underlie growth deficiency in dominant forms of OI. Our findings uncover the importance of HCs in the growth plate as target cells for therapeutic intervention and redefine OI as not only a bone disease, but also a cartilage disease.

## Methods

Supplemental Methods are available online with this article.

### Animals.

WT C57BL/6 mice (stock no. JAX000664), G610C OI mice on a C57BL/6 background (stock no. JAX007248) (36), Col1a1 2.3-GFP mice (stock no. JAX013134) (55), and Ai9 mice (stock no. JAX007909) (54) were obtained from The Jackson Laboratory. Col10a1-Cre–knock-in mice were produced in house (23, 43). These strains were maintained in the animal facility at The University of Maryland School of Medicine.

### EdU labeling and EdU detection with in situ hybridization for Col2a1.

To label proliferating cells in the growth plate, 5 mg/kg of EdU (Thermo Fisher Scientific) was i.p. injected into either C57BL/6 mice or G610C OI mice at 4 weeks of age. Tibiae were harvested 24 hours after EdU injection.

To evaluate EdU retention in the growth plate, 5 mg/kg of EdU was i.p. injected into mice in 2 consecutive days at 3 weeks of age, followed by tibia harvest 4 days after the last EdU injection.

The proximal half of harvested tibiae was fixed in 2% formaldehyde (Thermo Fisher Scientific) in sterile PBS at 4°C for 7 days, followed by dehydration in 10%, 20%, and 30% sucrose solution in sterile PBS containing 0.5% formaldehyde for a day each. Following embedding in SCMM mounting medium, frozen 10 μm–thick sections were made using an adhesive film (Cryofilm type IIIC; Section-Lab) following the Kawamoto’s Film method (77). The sections on the Cryofilm were then fixed with freshly prepared 2% formaldehyde in sterile PBS for 30 minutes at room temperature, followed by dehydration in 70% ethanol solution for a minute. After removing excessive ethanol, the sections on the Cryofilm were placed and attached to glass slides. A circle barrier around the sections was drawn with vacuum grease dissolved in spectroscopic-grade chloroform (MilliporeSigma). The edge of the film was covered with grease to keep the film attached to the glass slides. Once the grease dried, RNA in situ hybridization was performed following the RNAscope Multiplex Fluorescent Reagent Kit v2 User Manual (ACD) with some modifications. Briefly, the sections were incubated with RNAscope Hydrogen Peroxide for 10 minutes at room temperature. After washing with sterile PBS, the sections were digested with Protease III (ACD) at 40°C for 40 minutes followed by washing with sterile PBS. A probe for *Col2a1* (ACD) was applied to the sections and incubated at 40°C for 2 hours. After the serial amplification steps, the sections were incubated with fluorophore solution (Opal 570; Akoya Biosciences, 1:1000 dilution) at 40°C for 30 minutes. The sections were then stained for EdU using the Click-iT EdU Imaging Kit (Thermo Fisher Scientific), followed by counterstaining with DAPI (Thermo Fisher Scientific). The percentage of triple-positive cells for EdU, *Col2a1,* and DAPI in the entire growth plate (double-positive cells for *Col2a1* and DAPI) was calculated on at least 5 sections per bone using QuPath Bioimage Analysis software (University of Edinburgh) (78).

### 4PBA treatment.

In total, 0.4 mg of 4PBA (MilliporeSigma) diluted with sterile PBS or just sterile PBS as a control was daily administrated via i.p. injection. To examine the effects of 4PBA on osteoblastic transition and translocation of HCs, Col10a1-Cre;Ai9;Col1a1 2.3-GFP mice, and Col10a1-Cre;Ai9;Col1a1 2.3-GFP;G610C mice received the 4PBA administration from the age of 3 week for 10 days, followed by tissue harvest. Both femora and tibiae were fixed in 4% paraformaldehyde solution for μCT analysis and histological analysis.

For the treatment study, C57BL/6 WT mice and G610C OI mice were i.p. injected with 0.4 mg of 4PBA from the age of 3 weeks for 28 days, followed by i.p. injection of calcein (10 mg/kg body weight diluted with pH 7.4, 2% NaHCO_3_, MilliporeSigma) 2 and 12 days prior to euthanasia. One day after the last injection of 4PBA, femora and tibiae were harvested. Left femora from each mouse were fixed in 1% formaldehyde solution (Thermo Fisher Scientific) and stored at 4°C for dynamic histomorphometry. Right femora were wrapped with PBS-soaked gauze and stored at –20°C without fixation for μCT scan and for 4-point bending test. Right tibiae were fixed with 2.5% glutaraldehyde solution diluted with 0.1 M sodium cacodylate buffer at 4°C for a day, followed by fixation with 5% glutaraldehyde at 4°C for another day. The tibiae were further fixed and decalcified with 2.5% glutaraldehyde in pH 7.4, 0.1 M EDTA (MilliporeSigma) dissolved in 0.1 M sodium cacodylate buffer for a few days, followed by decalcification with 0.1 M EDTA without glutaraldehyde for 2 weeks. This decalcified tibia was used for electron microscopic analysis.

### Dynamic histomorphometry.

The diaphysis of left femora fixed with 1% formaldehyde solution was axially cut off at the distal portion of the third trochanter under a stereo microscope using a diamond cutting disc blade. The cut pieces of femoral diaphysis were washed in 100% ethanol, and remaining soft tissues were removed with micro forceps. The femoral diaphysis was embedded in a clear acrylic resin made by mixing 2.0 g of ClaroCit powder with 1300 μL of ClaroCit liquid (Struers). The axial cross section of the embedded femoral diaphysis was imaged with an EVOS Imaging System (Thermo Fisher Scientific) after polishing the surface with polishing disc (Dremel) and sandpaper (McMaster-Carr). The distance between 2 fluorescent lines from the first and second calcein injections on the posterolateral periosteal surface (mid-diaphysis) femur was measured on the acquired images using ZEN lite Digital Imaging software (Carl Zeiss Microscopy). Mineral apposition rate was calculated as the distance divided by 10, which is the time interval between the 2 injections.

### μCT analysis.

The stored right femora were scanned in PBS with SkyScan 1174 (Bruker) at a 0.6° increment angle with averaging 2 frames using a 50 kV/80 μA x-ray source with a 0.2 mm Al filter and a beam flattener to reduce beam hardening artifacts. The images were reconstructed at either a 6.5 μm or 13.6 μm isotropic voxel size for trabecular and cortical analyses, and bone length measurement, respectively. Cortical regions of interest (ROI) were centered in the midpoint between the middle of the distal femoral growth plate and the middle of the third trochanter, with thickness of 10% of the total femoral length (from the femoral head to the distal femoral condyle). Distal femoral trabecular ROI began proximally from 5.5% of the total femoral length, offset from the middle of the distal femoral growth plate with thickness of 10% of the total femoral length. Parameters including bone length, T. BMD, T. BV/TV, Tb. Th, Tb. N, C. TMD, C. Th, C. Ar, and C. Pm were quantified using CTAn software (Bruker).

### Four-point bending.

Following μCT, femora were loaded to failure in 4-point bending using an ElectroForce 5500 (TA Instruments). The femora were placed on the specimen rig with the posterior surface oriented under tension at a load rate of 0.005 mm/s. The distances of the upper and lower supports were 6.2 mm and 2.0 mm, respectively. Force and vertical displacement were acquired with WinTest software (TA Instruments).

### Double labeling assay.

To visualize mineralization in the hypertrophic zone and the primary spongiosa, alizarin complexone (MilliporeSigma; 30 mg/kg of body weight) and calcein (3 mg/kg of body weight) were s.c. injected into 3-week-old G610C OI mice and their WT littermates 48 hours and 1 hour prior to euthanasia, respectively. Tibiae were harvested and fixed in 4% paraformaldehyde solution for 2 days. These fixed tibiae were then gradually dehydrated in 10%, 20%, and 30% sucrose solution in PBS for a day each, followed by embedding in SCMM mounting medium (77). Frozen 5 μm–thick sections were made using an adhesive film (Cryofilm type IIIC; Section-Lab) following the Kawamoto’s Film method (77).

### Histology.

Harvested bones were fixed in 4% paraformaldehyde solution for 2 days, followed by decalcification with 15% EDTA in PBS at 4°C for 2–3 weeks. Bones were dehydrated with 10%, 20%, and 30% sucrose solution for a day each, before being embedded in OCT compound (Thermo Fisher Scientific). To detect endogenous fluorescent proteins such as tdTomato and GFP, 5 μm–thick frozen sections were mounted with an antifade reagent containing DAPI. All images were acquired either with an Axiocam MRm camera using AxioVision 4.5SP1 software on Zeiss Axio Imager A1 (Carl Zeiss) or with BZ-X700 using BZ-H4A software (Keyence). The number of green, red, and yellow cells in the trabecular areas (up to 500 μm from the growth plate) were calculated on at least 5 sections per bone.

### Electron microscopy.

The proximal epiphysis and metaphysis, including the growth plate, were dissected from the above decalcified tibia and fixed in 2% paraformaldehyde and 2.5% glutaraldehyde in 0.1 M PIPES buffer (pH 7.4) for 1 hour. Following fixation, bones were rinsed with 0.1 M PIPES, quenched with 50 mM Gly in 0.1 M PIPES buffer (pH 7) for 15 minutes, and washed again with 0.1 M PIPES buffer, followed by postfixation in 1% (w/v) osmium tetroxide, 0.75% ferrocyanide in 0.1 M PIPES buffer at 4°C for 60 minutes. After osmication, bones were washed with water, stained with 1% (w/v) uranyl acetate in water for 60 minutes, and dehydrated using serial graded ethanol solution including 30%, 50%, 70%, 90%, and 100% ethanol and 2 changes of 100% acetone. The specimens were infiltrated and embedded in araldite resin (Electron Microscopy Sciences) following manufacturer’s recommendation. Ultrathin sections about 70 nm in thickness were cut on a Leica UC6 ultramicrotome (Leica Microsystems Inc.) and collected onto formvar film–coated Synaptek notchdot grids (Electron Microscopy Sciences) and examined in a Tecnai T12 transmission electron microscope (Thermo Fisher Scientific) operated at 80 keV. Digital images were acquired by using a bottom-mount CCD camera (Advanced Microscopy Techniques) and AMT600 software. ER thickness was measured at 500 nm intervals in 5–12 HCs per growth plate (9–199 measurements per cell) or in 5–9 trabecular osteoblasts per bone (21–163 measurements per cell) on acquired images, as previously described (37).

### Chondrocyte pellet culture and quantitative PCR (qPCR).

Epiphyseal cartilage was dissected from humeri and femora of day 4 neonatal G610C OI mice. Primary chondrocytes were isolated by digesting the cartilage with collagenase, as previously described (79, 80). In total, 5 × 10^5^ chondrocytes were pelleted in a 0.5 mL tube by centrifugation at 400*g* for 5 minutes and cultured in DMEM/F12 with L-glutamine (Corning Life Sciences) supplemented with ascorbic acid (50 μg/mL) and 10% FBS (37). Following 3 weeks of culture, pellets were treated with or without 5 mM of 4PBA overnight. After washing the pellets with cold sterile PBS twice, the pellets were disrupted in RLT buffer containing β-mercaptoethanol using a mortar and pestle (Takara Bio Inc.) and homogenized with QIAshredder (QIAGEN). RNA was isolated from the lysate with a RNAeasy Micro Kit according to the manufacturer’s instruction (QIAGEN). Reverse transcription was performed with a High-Capacity cDNA Reverse Transcription Kit (Thermo Fisher Scientific). qPCR was performed on QuantStudio 6 Flex Real-Time PCR System (Thermo Fisher Scientific) using TaqMan Gene Expression Assays for *Gapdh*, *Col10a1*, *Alp*, and *Ibsp*.

### Statistics.

Statistical analysis was performed in SigmaPlot 13 software package (Systat). All groups of data were checked for normality (Kolmogorov-Smirnov test) and equal variance (Brown-Forsythe test). Comparisons of mean values between normally distributed groups were performed with 2-tailed Student’s *t* test or 2-way ANOVA with Holm-Sidak post hoc analysis, as appropriate and indicated in figure legends. Mann-Whitney *U* test was used whenever the normality test failed. Heteroscedastic, 2-tailed Student’s *t* test was used whenever the equal variance test failed. No *P* value adjustments for multiple comparisons were performed, since null hypotheses statistically tested in this study did not allow for type I error inflation. *P* < 0.05 was considered statistically significant.

### Study approval.

All animal work was conducted in accordance with protocols approved by the IACUC of the University of Maryland.

## Author contributions

ALS, KJW, AS, MEI, MI, SL, and SO conducted experiments and acquired data. ALS, KJW, AS, SL, and SO contributed to data analysis. ALS, KJW, AS, SL, and SO developed experimental methods. MEI, TK, MI, SL, and SO designed the experiments, designed the study concept, and contributed to data interpretation. KSEC provided reagents and discussed the data. ALS, KSEC, SL, and SO contributed to writing the manuscript.

## Supplementary Material

Supplemental data

## Version 1 01/06/2022

In-Press Preview

## Version 2 02/08/2022

Electronic publication
