# Supplementary material for: 4PBA reduces growth deficiency in osteogenesis imperfecta by enhancing transition of hypertrophic chondrocytes to osteoblasts
Source: JCI Insight. 2022 Feb 8;7(3):e149636. doi: 10.1172/jci.insight.149636 (PMC8855815; doi:10.1172/jci.insight.149636)
Supplement: Supplemental data [file jciinsight-7-149636-s100.pdf]

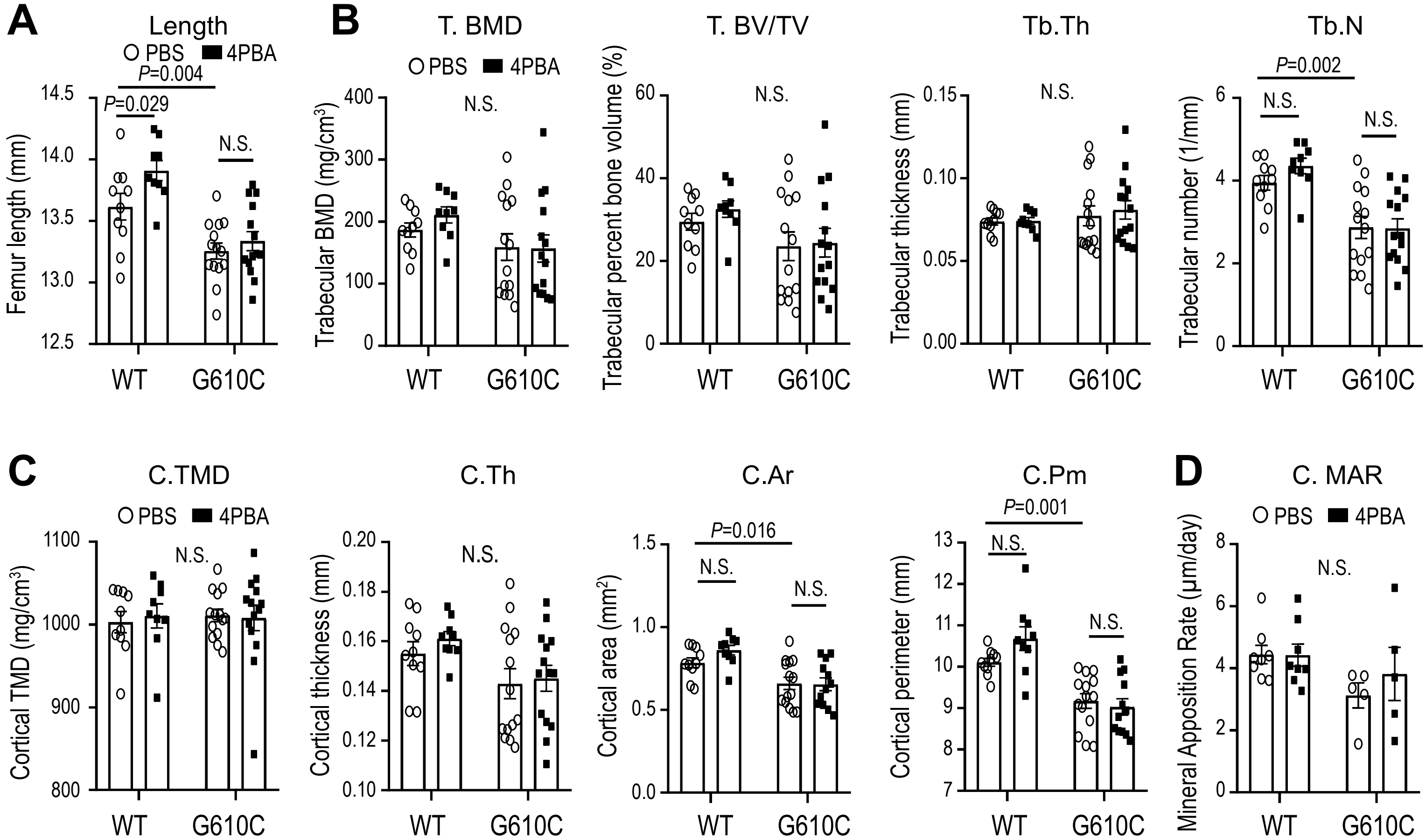

Supplemental Figure 1. Femur geometry (μCT) in male G610C OI mice and male wild type littermates (WT) treated by daily injections of PBS (circle dots) or 0.4 mg 4PBA in PBS (filled square dots) for 4 weeks (WT+PBS: n=10, WT+4PBA: n=9, G610C+PBS: n=14, G610C+4PBA: n=14). (**A**) Femur length (2-way ANOVA, treatment-genotype interaction $P > 0.1$). (**B**) Trabecular bone mineral density (T. BMD; 2-way ANOVA, treatment-genotype interaction $P > 0.1$), trabecular bone volume fraction (T. BV/TV; t-test), trabecular thickness (Tb. Th; U-test), and trabecular number (Tb. N; 2-way ANOVA, treatment-genotype interaction $P > 0.1$). (**C**) Cortical tissue mineral density (C. TMD; 2-way ANOVA, treatment-genotype interaction $P > 0.1$), cortical thickness (C. Th; 2-way ANOVA, treatment-genotype interaction $P > 0.1$), cross-sectional area (C. Ar; 2-way ANOVA, treatment-genotype interaction $P > 0.1$) and perimeter (C. Pm; 2-way ANOVA, treatment-genotype interaction $0.05 < P < 0.1$). (**D**) Mineral apposition rate (MAR) at posterolateral periosteal surface (mid-diaphysis) per day (2-way ANOVA, treatment-genotype interaction $P > 0.1$). Bar charts show mean ± SEM. N.S. = not significant. U- or t- tests were used instead of 2-way ANOVA in **B** because of failure of normality or equal variance tests, respectively.

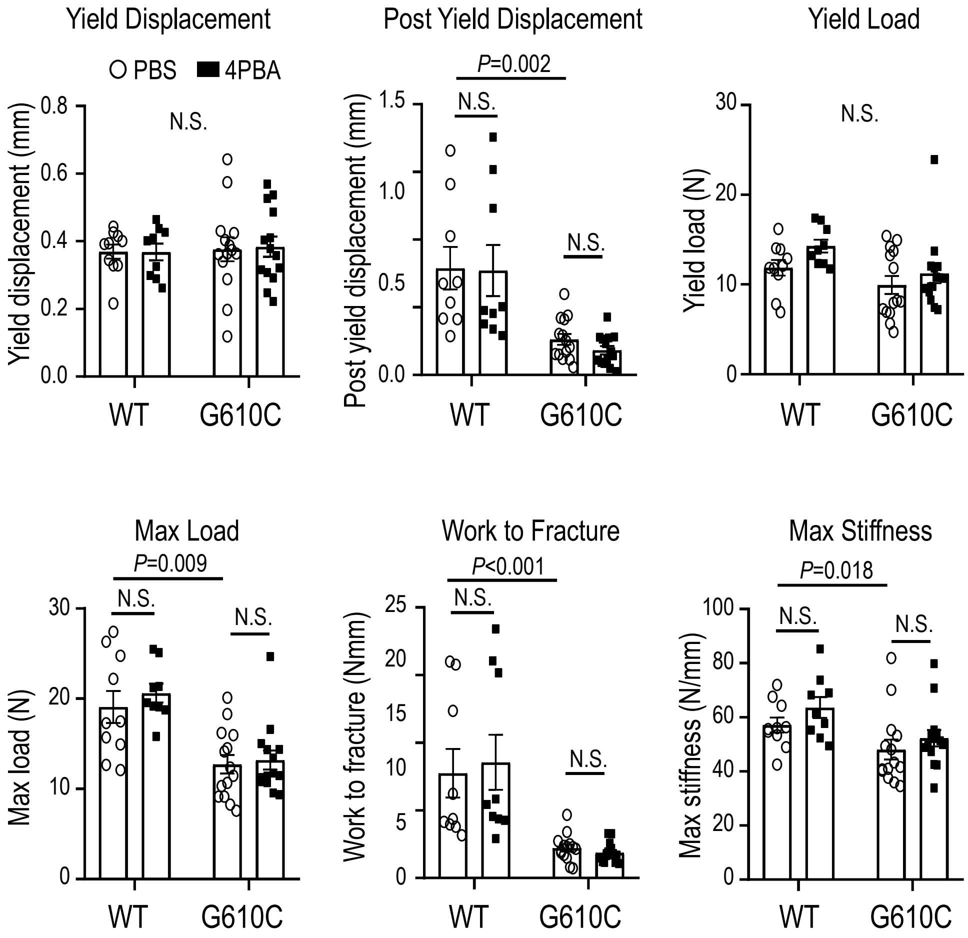

Supplemental Figure 2. Effect of daily injections of PBS (circle dots) or 0.4 mg 4PBA in PBS (filled square dots) for 4 weeks on biomechanical parameters of the same femora as in **Supplemental Figure 1** (4-point bending test at mid-shaft). Bar charts show mean ± SEM. N.S.= not significant. 2-way ANOVA was used for Yield Load and Yield Displacement. U-test was used for Max Load, Work to Fracture and Max Stiffness (failed normality test).

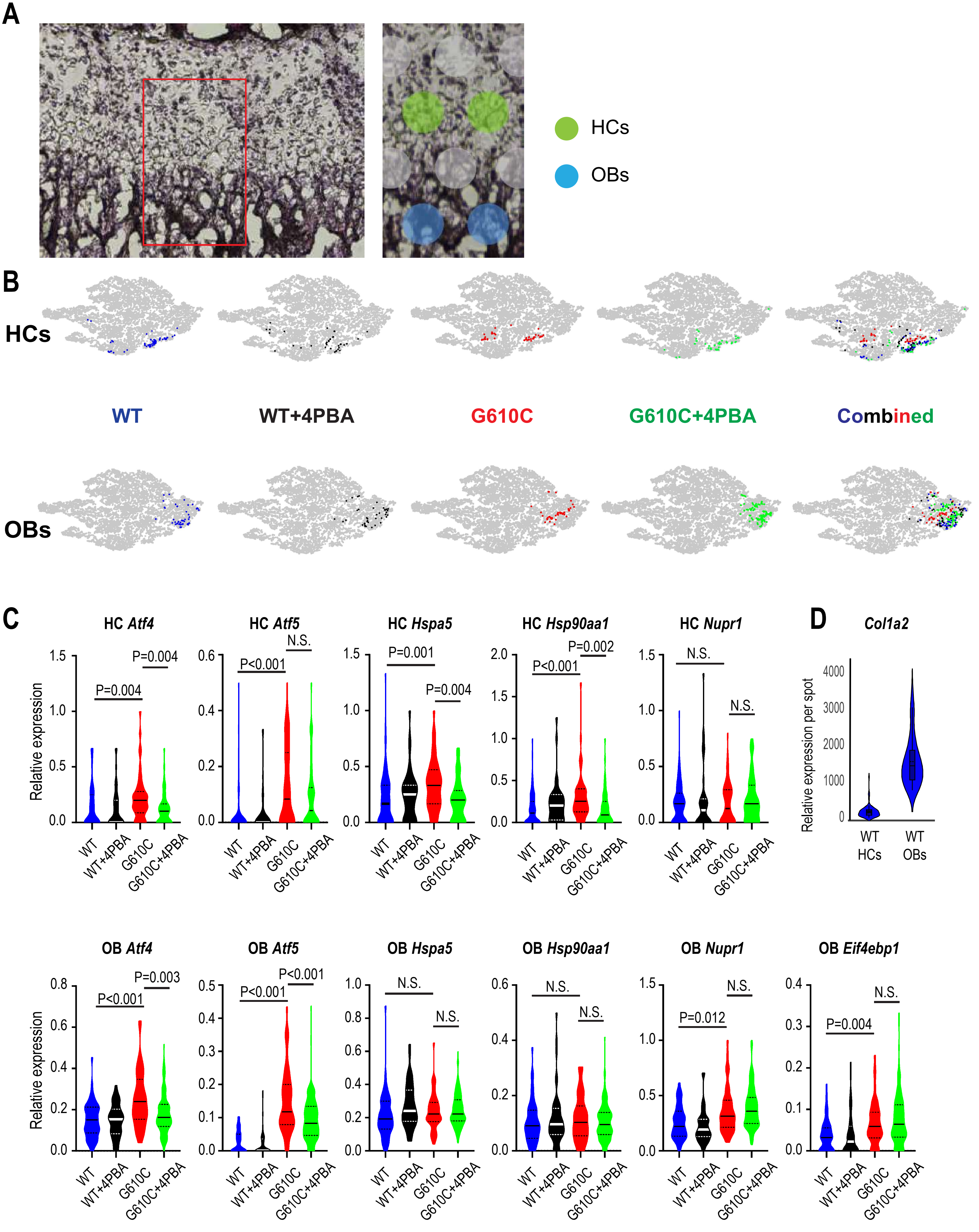
A
HCs
OBs
B
HCs
WT
WT+4PBA
G610C
G610C+4PBA
Combined
OBs
C
HC Atf4
HC Atf5
HC Hspa5
HC Hsp90aa1
HC Nupr1
Relative expression
P=0.004
P=0.004
P<0.001
N.S.
P=0.001
P=0.004
P<0.001
P=0.002
N.S.
N.S.
D
Col1a2
Relative expression per spot
WT HCs
WT OBs
OB Atf4
OB Atf5
OB Hspa5
OB Hsp90aa1
OB Nupr1
OB Eif4ebp1
P<0.001
P=0.003
P<0.001
P<0.001
N.S.
N.S.
N.S.
N.S.
P=0.012
N.S.
P=0.004
N.S.
WT
WT+4PBA
G610C
G610C+4PBA

Supplemental Figure 3. Effects of G610C mutation and 10-day 4PBA treatment on the transcriptome of hypertrophic chondrocytes (HCs) and mature trabecular osteoblasts (OBs) in the tibia of 4.5-week-old mice. (**A**) Spatially resolved transcriptome (SRT) assay for HCs and OBs in the tibial growth plate. 10 µm cryosections of the proximal growth plate stained with H&E (hematoxylin and eosin, left image) were deposited onto a Visium™ (10X Genomics) slide coated with oligonucleotides that capture mRNA from permeabilized tissue at 55 µm round spots (overlaid onto H&E staining in the zoomed panel). Within each spot, oligonucleotides have a unique sequence, identifying cDNA from each spot containing 1-10 cells after reverse transcription. Spots enriched with HCs (green) and OBs (blue) were selected based on their locations within the growth plate and expression of marker genes (see Methods below). (**B**) T-distributed stochastic neighbor embedding (t-SNE) maps of HC-enriched and OB-enriched spot transcriptomes in wild type (WT) and G610C mice treated with either PBS (control) or 4PBA injections. Each dot in the t-SNE maps represents a spot on the sections. Spots with similar transcriptomes are clustered closely. Cells of the same type might be located in different clusters because of the effects of different cell origins or environments. Therefore, only the overall effects of 4PBA can be interpreted. (**C**) Effects of genotype and 4PBA treatment on expression of representative transcripts of well-known cell stress genes (e.g, see Gene Ontology and similar databases). To illustrate the difference between HCs and OBs, we selected 2 genes detected as significantly upregulated both in G610C HCs and G610C OBs (*Atf4* and its *Atf5* paralogue), 2 genes detected as upregulated in HCs but not in OBs (*Hspa5* and *Hsp90aa1*), and 2 genes detected as upregulated in OBs but not in HCs (*Nupr1* and *Eif4ebp1*). Note that only highly expressed genes could be reliably measured by Visium SRT. (**D**) Relative expression (normalized counts per spot) of *Col1a2* by HCs and OBs (WT mouse treated with PBS). Violin plots show median ± quartiles. N.S.=not significant.

Results: We observed transcriptome rescue by 4PBA in most HCs, consistent with the rescue of the HC to OB transition and bone growth (top of panel B, good overlap of most blue and green spots). In contrast, 4PBA appeared to rescue the transcriptome of only a few OBs, consistent with its much weaker effect on OB function and bone strength (bottom of panel B, poor overlap of blue and green spots). This difference could be related to different types of cell stress response to the G610C mutation in HCs and OBs.

Indeed, activation of different subsets of key cell stress genes in HCs (*Hspa5*, *Hsp90aa1*, but not *Nupr1*) and OBs (*Nupr1*, *Eif4ebp1*, but not *Hspa5* and *Hsp90aa1*) indicates that different types of cell stress responses to the mutation are induced in HCs and OBs (panel C). At the same time, activation of *Atf4* and *Atf5* in HCs and OBs confirms downstream effects of cell stress on these stress-induced regulators of protein translation in both cell populations. Activation of UPR master regulator *Hspa5* (BIP) and *Hsp90aa1* in HCs but not in OBs was reproduced in 3 different SRT experiments. It is also consistent with our previous findings (17, 37). This UPR might be a secondary response to ER disruption causing misfolding of globular proteins, while the primary misfolding of mutant type I collagen disrupts the ER without sequestering BIP and triggering primary UPR (72). More pronounced secondary UPR in HCs compared to osteoblasts could be related to lower expression of type I collagen and higher expression of globular ECM proteins by HCs (the amount of *Col1a2* mRNA in HC-enriched spots is ~10 times lower than that in OB-enriched spots, panel D). The difference in the cell stress response between HCs and OBs might contribute to the weaker effect of 4PBA on OB transcriptome and function.

Note that full analysis of the Visium SRT findings and comparison with other RNA sequencing and *in situ* hybridization assays will be presented in a separate study of cell stress pathways in G610C HCs and OBs, which is beyond the scope of the present paper. While Visium SRT assay is well suited for supporting different types of cell stress and different effects of 4PBA on HCs and OBs, combining it with the other assays is important for a more detailed analysis of cell stress pathways. For instance, the high expression of *Hspa5* allowed us to use Visium SRT for confirming its activation in G610C HCs but not OBs. At the same time, the limited sensitivity of this assay could prevent us from detecting some other differentially regulated cell stress genes with lower expression and thereby identifying specific UPR pathways activated in HCs.

***Methods**:* Visium SRT assay was optimized and performed as recommended by 10X Genomics. Briefly, 2 WT and 2 G610C littermates were treated daily with 4PBA (0.4mg) or PBS for 10 days starting at 3 weeks after birth. The right tibia was rapidly harvested and freshly frozen in OCT (Sakura Finetek). 7-8 cryosections (10 µm-thickness) of the proximal growth plate and adjacent tissue from each of the animals were placed in each of the 4 capture areas within a Visium slide, fixed with methanol, stained with H&E, imaged, and permeabilized for 45 min to allow for mRNA binding to slide oligonucleotides. Subsequent cDNA synthesis, amplification, and purification were performed as described in the Visium kit for frozen sections (10X Genomics). Library construction and cDNA sequencing followed by genome alignment and data aggregation with Space Ranger software (10X Genomics) were performed by the Molecular Genomics Core of NICHD. The transcriptomes were mapped and extracted from the data set with Loupe Browser 5.0 (10X Genomics). *Actg1* was used as a housekeeping gene for calculating normalized relative expression. In addition to the location, HC-enriched spots were identified based on the relative expression of *Col10a1*, *Col2a1*, *Acan*, and *Col1a1*. OB-enriched spots were identified based on *Col1a1, Col10a1*, *Col2a1*, and *Ibsp* expression. Overall, 3 experiments were performed. In two of them, sections from PBS-treated WT animals were lost or damaged during processing, yet all 3 experiments produced consistent results for other animals and for the effect of 4PBA treatment on G610C mice. Given the cost of the assay, no further experiments were performed. This figure shows the experiment with both WT controls, in which 40-70 HC-enriched and 40-100 OB-enriched spots were analyzed for each animal. The statistical significance (P-values) was analyzed with 2-way ANOVA followed by a pairwise *U*-test since the normality test failed. Each Visium slide spot was considered a biological replicate.
